# Supplementary material for: MetaQTL: a package of new computational methods for the meta-analysis of QTL mapping experiments
Source: BMC Bioinformatics. 2007 Feb 8;8:49. doi: 10.1186/1471-2105-8-49 (PMC1808479; doi:10.1186/1471-2105-8-49)
Supplement: Additional File 7 — MetaQTL Package : jar file and tutorial. This Zip archive contains both the MetaQTL JAR file and the files of the tutorial. [file 1471-2105-8-49-S7.zip › org.inra.metaqtl/doc/org/metaqtl/IMetaQtlConstants.html]

IMetaQtlConstants


|  |  |  |
| --- | --- | --- |
| **Overview** \| **Package** \| **Class** \| **Use** \| **Tree** \| **Deprecated** \| **Index** \| **Help** | |  |
| **PREV CLASS**   **NEXT CLASS** | **FRAMES**    **NO FRAMES**     **All Classes** |  |
| SUMMARY: NESTED \| FIELD \| CONSTR \| METHOD | DETAIL: FIELD \| CONSTR \| METHOD |  |


---


## org.metaqtl Interface IMetaQtlConstants

---

``` public interface IMetaQtlConstants ```

This interface defines some global constants which are used
by several classes of the package.

---

| **Field Summary** | |
| --- | --- |
| `static int` | `CI_MISS_IGNORE`             MSD Mode : ignore QTL with missing standard deviation. |
| `static int` | `CI_MISS_IMPUTATION`             MSD Mode : replace missing standard deviation by the mean of the observed ones. |
| `static int` | `CI_MODE_AVAILABLE`             SD Mode : use the available information to compute the standard deviation. |
| `static int` | `CI_MODE_LOD`             SD Mode : use only the confidence interval information if defined to compute the standard deviation. |
| `static int` | `CI_MODE_MAXIMAL`             SD Mode : compute the standard deviation using both lod support and rsquare information (if available) and then choose the maximal standard-deviation. |
| `static int` | `CI_MODE_RSQUARE`             SD Mode : use only the rsquare information if defined to compute the standard deviation. |
| `static int` | `MAPPING_FUNCTION_HALDANE`             Integer code for haldane mapping function |
| `static int` | `MAPPING_FUNCTION_KOSAMBI`             Integer code for kosambi mapping function |
| `static int` | `MAPPING_FUNCTION_UNKNOWN`             Integer code for not defined mapping function |
| `static int` | `MAPPING_UNIT_CM`             Integer code for centi morgan mapping unit |
| `static int` | `MAPPING_UNIT_M`             Integer code for morgan mapping unit |
| `static int` | `MAPPING_UNIT_UNKNOWN`             Integer code for unknown mapping unit |
| `static double` | `TINY_MAPPING_DISTANCE`             The minimal distance in morgan |
| `static double` | `TINY_MAPPING_VARIANCE`             the minimal variance in morgan |
| `static java.lang.String` | `VERSION`             The version of the package. |

**Field Detail**

### VERSION

```
static final java.lang.String VERSION
```

:   The version of the package.

    **See Also:**: Constant Field Values

---


### MAPPING\_FUNCTION\_UNKNOWN

```
static final int MAPPING_FUNCTION_UNKNOWN
```

:   Integer code for not defined mapping function

    **See Also:**: Constant Field Values

---


### MAPPING\_FUNCTION\_HALDANE

```
static final int MAPPING_FUNCTION_HALDANE
```

:   Integer code for haldane mapping function

    **See Also:**: Constant Field Values

---


### MAPPING\_FUNCTION\_KOSAMBI

```
static final int MAPPING_FUNCTION_KOSAMBI
```

:   Integer code for kosambi mapping function

    **See Also:**: Constant Field Values

---


### MAPPING\_UNIT\_UNKNOWN

```
static final int MAPPING_UNIT_UNKNOWN
```

:   Integer code for unknown mapping unit

    **See Also:**: Constant Field Values

---


### MAPPING\_UNIT\_M

```
static final int MAPPING_UNIT_M
```

:   Integer code for morgan mapping unit

    **See Also:**: Constant Field Values

---


### MAPPING\_UNIT\_CM

```
static final int MAPPING_UNIT_CM
```

:   Integer code for centi morgan mapping unit

    **See Also:**: Constant Field Values

---


### TINY\_MAPPING\_DISTANCE

```
static final double TINY_MAPPING_DISTANCE
```

:   The minimal distance in morgan

    **See Also:**: Constant Field Values

---


### TINY\_MAPPING\_VARIANCE

```
static final double TINY_MAPPING_VARIANCE
```

:   the minimal variance in morgan

    **See Also:**: Constant Field Values

---


### CI\_MODE\_AVAILABLE

```
static final int CI_MODE_AVAILABLE
```

:   SD Mode : use the available information to compute the
    standard deviation.

    **See Also:**: Constant Field Values

---


### CI\_MODE\_LOD

```
static final int CI_MODE_LOD
```

:   SD Mode : use only the confidence interval information
    if defined to compute the standard deviation.

    **See Also:**: Constant Field Values

---


### CI\_MODE\_RSQUARE

```
static final int CI_MODE_RSQUARE
```

:   SD Mode : use only the rsquare information
    if defined to compute the standard deviation.

    **See Also:**: Constant Field Values

---


### CI\_MODE\_MAXIMAL

```
static final int CI_MODE_MAXIMAL
```

:   SD Mode : compute the standard deviation using
    both lod support and rsquare information (if available)
    and then choose the maximal standard-deviation.

    **See Also:**: Constant Field Values

---


### CI\_MISS\_IMPUTATION

```
static final int CI_MISS_IMPUTATION
```

:   MSD Mode : replace missing standard deviation
    by the mean of the observed ones.

    **See Also:**: Constant Field Values

---


### CI\_MISS\_IGNORE

```
static final int CI_MISS_IGNORE
```

:   MSD Mode : ignore QTL with missing standard deviation.

    **See Also:**: Constant Field Values


---


|  |  |  |
| --- | --- | --- |
| **Overview** \| **Package** \| **Class** \| **Use** \| **Tree** \| **Deprecated** \| **Index** \| **Help** | |  |
| **PREV CLASS**   **NEXT CLASS** | **FRAMES**    **NO FRAMES**     **All Classes** |  |
| SUMMARY: NESTED \| FIELD \| CONSTR \| METHOD | DETAIL: FIELD \| CONSTR \| METHOD |  |


---
